# Are Your Employees Hopeful at Work? The Influence of Female Leadership, Gender Diversity and Inclusion Climate on Japanese Employees’ Hope

**DOI:** 10.3389/fpsyg.2022.936811

**Published:** 2022-07-18

**Authors:** Soyeon Kim

**Affiliations:** Faculty of International Social Sciences, Gakushuin University, Tokyo, Japan

**Keywords:** female leadership, ambidextrous leadership, gender diversity and inclusion climate, hope at work, Japan, employees’ gender

## Abstract

There are two well-known truths about Japan: one is that Japan is one of the most advanced economies, which takes pride in its highly advanced technology, social infrastructure and system; the other is that Japan ranks lowest at women’s social participation among Organization for Economic Co-operation and Development countries. Even though the Japanese government has initiated programs to promote female participation and advancement in society, these initiatives have not yet borne remarkable fruit. This study intends to address this issue by investigating the effectiveness of female leadership in Japan, specifically its effect on organizations’ gender diversity and inclusion (D&I) climate and employees’ task-related positive attitudes. Synthesizing social information processing theory and social identity theory, the study examines 306 Japanese employees working with their female supervisors in medium- and large-sized manufacturing companies. The findings show that female ambidextrous leadership contributes to shape and strengthen a gender D&I climate and ultimately enhances employees’ hope on their work. In addition, the positive effect of a gender D&I climate on employees’ hope is the same for all employees regardless of gender. The findings clarify the role of female leadership and the underlying psychological mechanism through which female leadership influences employees’ positive work attitudes. This first empirical study in Japan contributes to the research on female leadership and D&I management.

## Introduction

Responding to social awareness and the movement toward diversity, workforce diversity and inclusion (D&I) has become a significant topic in human resource management (Farndale et al., 2015). As part of sustainable development goals, ensuring fairness and embracing diversity in managing employees have become imperative for business. The younger generation is particularly conscious of corporate social responsibilities that include companies’ endeavors regarding the fair and equal treatment of employees (Alonso-Almeida and Llach, 2019). Building a corporate D&I brand identity by establishing and implementing D&I practices is becoming more important in terms of attracting talent (Jonsen et al., 2021). Despite the increased focus on D&I and its importance, research on the topic is in the embryonic stage, thus its effective formulas and practical implementation in companies are not clearly disclosed yet.

Generally, workforce diversity encompasses demographic aspects including gender, age, race, disability, and sexual orientation. Of these, gender diversity is an immediate issue in Japan (Ichikohji, 2016; Namakura, 2017). Since the start of the millennium, there has been practical discussion about embracing a female workforce and the potential economic benefits of female participation in Japan. In 2014, the Abe administration adopted the idea of Womenomics^1^ and initiated nationwide programs to support women’s participation and advancement in Japan. Such attention and initiatives on gender D&I have drawn some progress. The rate of female workers aged 15–64 has gradually increased from 63.63% in 2012 to 72.77% in 2019 (The World Bank, 2021). However, this quantitative increase in female participation has not come with qualitative growth in women’s advancement. Japanese women continue to suffer from disadvantages in rewards and career development in companies. They are paid 32% less than their male counterparts and face obstacles to promotion to senior positions. Women occupy only 12.6% of corporate board positions in listed companies in 2021, which is far below the average of OECD countries (28%) and very low compared with that in other advanced economies, such as Canada (32.9%), France (45.3%), and the United Kingdom (37.8%) (OECD, 2022).

Against this background, this study deals with gender D&I in the business sector of Japan. Specifically, the study focuses on female leadership and investigates its effectiveness on gender D&I climate and employees’ hope in Japan. Synthesizing ambidextrous leadership theory (Rosing et al., 2011), social information processing theory (Salancik and Pfeffer, 1978), and social identity theory (Tajfel and Turner, 1986), the study clarifies how female ambidextrous leadership helps shape and strengthen a diversity climate and thus increases employees’ hope on their work. Even though studies on female leadership have been gradually increasing, these have been confined to the expected advantages of female leadership (Eagly and Carli, 2003; Stoker et al., 2012), the effectiveness of such leadership (Szymanska and Rubin, 2018), and female leaders in specific sectors and contexts (Girdauskiene and Eyvazzade, 2015). These explorations have endeavored to illuminate why more women deserve assignment to management positions and provide evidence of the benefits that can be anticipated from the promotion of women in organizations. They have added value to female leadership research, but they have not clearly answered the fundamental question of how female leaders should behave. That is, the most effective female leadership qualities and their effects have yet to be determined. To cast light on this issue, the current work adopted the concept of ambidextrous leadership in investigating female leadership qualities and the mechanism expected to underlie it.

This study also focused on employees’ hope, a component of psychological capital (Luthans et al., 2007), which pertains to a positive psychological status that is important in determining individuals’ work attitudes, behaviors and task performance (Avey et al., 2011; Alessandri et al., 2018). Hope toward work, as an element constituting psychological capital, is related to work motivation and thus holds particular importance for the achievement of work goals. Hope among employees increases positive energy at work and directs them to engage in productive and creative job activities, thereby elevating the possibility of success (Rego et al., 2009; Anwar et al., 2019). Hopeful employees are also intrinsically motivated and perseverant, so when they face obstacles and problems in the workplace, they endeavor to take a positive stance and find a different pathway, which in turn helps them overcome risks and generate favorable outcomes.

Social information processing theory posits that an organization’s socio-environmental factors influence the shaping of employees’ positive psychological states (Salancik and Pfeffer, 1978). Employees’ information processing and appraisal of their work environment determines their levels of psychological capital (Luthans et al., 2007; Srivastava and Maurya, 2017), which is also significantly determined by the leadership of supervisors, among various organizational factors (McMurray et al., 2010; Rego et al., 2012). Notwithstanding the insights provided by previous research, however, the effects of leadership from female supervisors on employees’ positive psychology have rarely been studied. This paucity is particularly serious in Japan. Some academic attempts have been made to inquire into female leaders in Japan, but these efforts have been limited to discussions of the necessity or urgency of cultivating female entrepreneurs and female leaders in business (Miyake, 2015; Takada, 2009). No study has addressed the leadership characteristics required for women to take on this position, the effectiveness of female leadership, and its potential influence on employees in the business sector in Japan.

Correspondingly, the present work was intended to identify effective female leadership styles and their influence on employees’ perceptions regarding the diversity climate of organizations and hope toward work. Considering the flexible, balanced, and open characteristics of female leadership (Girdauskiene and Eyvazzade, 2015; Sueda et al., 2020), this study centered on ambidextrous leadership among women and its effects on employees in Japanese manufacturing companies. The findings can contribute to the development of female leadership studies by shedding light on unanswered questions and doubts related to female leadership’s expected effects in Japanese organizations. By providing empirical evidence from the analysis of survey responses, this study offers practical advice and solutions to companies’ decision makers who are unconvinced about women’s leadership abilities, thereby aiding companies in facilitating the implementation of effective gender D&I management. This research is a timely initiative that is expected to add value to current theoretical studies, businesses, and society.

## Literature Review

### Gender D&I Management in Japan

The concept of D&I is no longer new; in fact, it has been advocated globally. D&I in companies has been discussed by critics of serious and prevalent social problems such as unfair treatment, stereotyping, prejudice, social discrimination, and social stratification due to demographic differences among individuals (Shore et al., 2009). Individuals’ demographic features are not chosen, but naturally given; however, these factors play roles, explicitly or implicitly, in terms of determining the boundaries of individuals’ social activities, such as job choice, wage range, chance of promotion, and so forth. Such restrictions on free will and choices discourage individuals, hindering their growth and development, which ultimately leads individuals’ counterproductive behaviors, increasing problems and costs. Recently, stakeholders involved in companies’ business activities are more concerned about companies’ D&I practices (Alonso-Almeida and Llach, 2019; Jonsen et al., 2021). They request fair treatment of employees maintaining diversity and consequently, D&I management has become imperative in the business world (Madera et al., 2018; Jonsen et al., 2021). This implies that companies should take a strategic approach in D&I by investing in building a D&I brand image as a part of impressment management to attract their stakeholders.

D&I management in Japanese companies is not progressive compared to the one in other advance economies. This stagnancy in gender D&I management is partly because of Japan’s distinctive cultural values and societal–contextual characteristics. In pursuing group harmony and not causing noise or conflict, unanimous decision making based on the so-called Ringi (稟議, the decision-making process in which a proposal or suggestion is reviewed and approved by all participants in a group) system is commonly implemented throughout Japanese society (Genzberger, 1994). Moreover, this tendency to maintain harmony in a group is made possible and is strengthened by the ethnic homogeneity of Japanese society. As of 2020, 97.71% of the population was Japanese, with only 2.19% being non-Japanese, mostly from Asian countries whose cultures have something in common with Japan. Current statistics (Statistics Bureau of Japan, 2022) indicate that people of an Asian nationality account for 84.12% of the total foreign population in Japan. The socio–contextual homogeneity of Japanese society strengthens its appreciation of the values of harmony and oneness and the need to avoid conflict situations. These values fundamentally collide with the values of individuality and variety that underlie D&I management in which conflict and discord are inevitable, so conflict situations should be encouraged from the outset to be solved not to be avoided. The contradictions cause a considerable gap between the founding values and the reality faced by Japan, thus delaying the comprehension, adoption, and implementation of D&I management in Japanese companies.

In Japan, gender D&I management has been gaining momentum since the start of the millennium due to the internal and external pressures. Japan’s population is aging, and the country is faced with a reduced workforce and a limited supply of young talent. This shortage is associated with the high possibility of the diminishing competitiveness of Japanese companies, which will put the Japanese economy at risk. This national demographic imbalance necessitates that companies embrace a more diverse workforce. Also, Japanese global firms are pressured to follow the global standards by assuring gender equality. Led by global Japanese companies, which are under high normative pressure to conform to advanced global standards in managing human resources, awareness of gender D&I management in Japan has increased. Since the enforcement of the act promoting female employment in 2015, large Japanese companies (with over 300 employees) have been obliged to make their gender diversity management plans public. Specifically, companies must report to the Ministry of Health, Labor, and Welfare about whether they provide equal opportunities to women and about the extent to which their organizations’ working environment is ready to embrace female workers. The act will be extended to medium-sized companies (with over 100 employees) in April 2022 (Ministry of Health, Labour, and Welfare, 2022). Due to this coercive and normative pressures from the world business, government, and the social need for diversity management, Japanese companies have become more engaged in devising and practicing gender D&I management. Thus, women’s social participation in Japan has been gradually increasing, showing the quantitative growth in number of female workers.

### Social Information Processing Theory and Female Leadership

According to social information processing theory, an individual is an “adaptive” entity (Salancik and Pfeffer, 1978). Individuals are influenced by the social environment in which they work, and therefore their attitudes and behaviors are determined by their perceptions and interpretations of the nature of the organizational environment. As Salancik and Pfeffer (1978, 226) state, “One can learn most about individual behavior by studying the informational and social environment within which that behavior occurs and to which it adapts.” This implies the importance of understanding the social context of organizations to understand and predict employees’ attitudes toward works. In particular, affective attitudes toward work are highly related with organizational characteristics (O’Reilly and Roberts, 1975). Of the various organizational factors that revolve around employees, leadership has an especially influential impact (Kim and Shin, 2019; Hu et al., 2020) due to the frequent and proximate interactions between employees and leaders. Leadership behaviors send a signal to employees about the values and direction the organization is pursuing and thus shape employees’ attitudes toward work. As a result, certain leadership behaviors can motivate employees and guide them toward achieving the organizational goals.

Female leaders are argued to have specific characteristics (Girdauskiene and Eyvazzade, 2015; Sueda et al., 2020). Sueda et al. (2020) studied female leaders in four different countries and found they have distinctive characteristics; for example, they have long-term views, set higher and broader vision, embrace the different ideas of others, and exhibit flexible behavior. Similarly, Girdauskiene and Eyvazzade (2015) found that female leaders are flexible and open minded and that these characteristics are very effective when managing people. Related research argues that female leaders are more transformational, communal, and relational than male leaders (Lipman-Blumen, 1992; Eagly and Carli, 2003) and further, researchers discovered that female leadership is as effective as male leadership (Eagly, 2007; Kim and Shin, 2017). Even if there is no specifically named leadership style that describes female leadership, previous studies have consistently argued that female leadership is characterized as being balanced, flexible, and open minded. Building on these findings, the present study focuses on ambidextrous leadership in investigating the effectiveness of female leadership in Japanese organizations.

### Female Ambidextrous Leadership, Gender D&I Climate, and Employees’ Hope

Rosing et al. (2011) define ambidextrous leadership as “the ability to foster both explorative and exploitative behaviors in followers by increasing or reducing variance in their behaviors and flexibly switching between those behaviors” (p. 957). The explorative behaviors of allowing and providing autonomy to employees and letting them generate and test new ideas are referred to as opening behaviors. The exploitative behaviors of managing and controlling employees by monitoring and evaluating their progress and achievements are referred to as closing behaviors. These two kinds of behaviors exhibited by ambidextrous leaders are argued to be complementary and to have an integrative effect on enhancing employees’ task performance by clarifying their role and increasing self-efficacy (Hu et al., 2020). As argued in the literature, ambidextrous leaders allow flexibility and autonomy to employees when they work, such relationship with ambidextrous leaders have employees feel confident and passionate regarding their work (Ma et al., 2019; Jiang et al., 2021). Thus, ambidextrous leaders help employees shape positive psychological state at work and such psychological capital of employees results in the increase of work performances (Zacher et al., 2016; Hu et al., 2020).

Given the proven positive effects of ambidextrous leadership on employees’ work attitudes, ambidextrous leadership of female leaders is expected to increase employees’ hope. As one component of psychological capital (Luthans et al., 2007), hope on work is defined as “a positive motivational state that is based on an interactively derived sense of successful (a) agency (goal-directed energy) and (b) pathways (planning to meet goals)” (Snyder et al., 1991, p. 287). Employees’ hope on work is achieved when they are clear about the work goals and at the same time when they have room to think about how to achieve these goals and to test alternative work paths (Snyder, 2000). Employees exert hopeful and positive energy at working when they are confident about what should be achieved and also their ideas and thoughts are appreciated. Female leaders’ ambidextrous leadership behaviors are open to individual different ideas and allow flexibility at work with clear guidelines about work goals and providing feedback. Such opening and closing behaviors flexibly exhibited by female leaders influence employees to be confident and positive at their work, which contributes to increasing their hope on work. Therefore, the following hypothesis is proposed:

**H1**. Female ambidextrous leadership positively influences employees’ hope on work.

However, if the gender of leaders is considered, that is, if ambidextrous leadership behaviors are exhibited by female managers, the underlying mechanisms through which female ambidextrous leadership affects employees’ work attitude can differ due to the distinctive information gained from such female leadership. According to social information processing theory (Salancik and Pfeffer, 1978), individuals recognize salient information, and their perception and interpretation of the information determines their work attitude. Because leader gender is salient for employees in less diverse organizations (Stoker et al., 2012), female ambidextrous leadership (a combination of “female leader” and “ambidextrous leadership”) sends a particular signal about the organization’s gender D&I climate; that is, how female workers are treated. Along with the existence of female leaders, their implementation of balanced leadership behaviors and flexibility in practicing exploitative and explorative behaviors signal organizations’ pro-gender D&I climate. As such, female ambidextrous leadership shapes employees’ perception that their organization treats employees fairly and has a favorable environment in which individuals’ different needs and values are supported through the implementation of inclusive policies and practices in pro-gender D&I climate. Such a perception improves employees’ organizational identity, thus motivating them to devote more energy to their work. This implies that female ambidextrous leadership helps create a gender D&I climate where fair treatment and valuing different ideas are encouraged and different ways of working are accepted in order to achieve employees’ work goals. Consequently, the flexible leadership behaviors of female leaders motivate employees to be positive and proactive in their work through their perception of pro-gender D&I climate. This suggests the potential mediating role of a gender D&I climate in the relationship between female ambidextrous leadership and employees’ hope on work. Therefore, the following hypothesis is proposed:

**H2.** Gender D&I climate mediates female ambidextrous leadership and employees’ hope on work.

The salient organizational information and its relative importance are different depending on individual social groups (McKay et al., 2008; Newman et al., 2018). According to social identity theory (Tajfel and Turner, 1986), people tend to seek an environment that is of immediate interest, and if they perceive that environment is supportive, its expected positive effect is relatively greater for them than for those in other social groups. Generally, minority groups are exposed to discrimination and experience unfair treatment in their organizations; therefore, they are more sensitive to D&I climate, and their perceptions in this regard have a greater effect on their attitudes and performance compared to majority groups (McKay et al., 2008; Newman et al., 2018); Newman et al. (2018) found that diversity climate increases employees’ psychological capital, thus elevating organizational commitment and reducing turnover intentions. However, this positive effect of diversity climate is moderated by ethnic identity. In studying sales employees in the US, McKay et al. (2008) found that diversity climate has considerable effects on employees belonging to ethnic minorities. Such climate enhances the employees’ perceptions of fair and equal treatment of human resources in organizations, thus reducing discrimination and conflicts among ethnically different groups and enhancing sales performance. Companies’ gender D&I climate denotes employees’ perceptions of companies’ structured and implemented practices and policies regarding the treatment of female employees. The organizational information of gender D&I is more salient for female employees who are generally under-represented and less well treated compared to male employees. Therefore, companies’ efforts in supporting and embracing female employees will be more important to female employees and will enhance their organizational identity and have a greater positive effect on their motivational and positive state at work. Based on this reasoning, the following hypothesis is proposed:

**H3.** The positive link between gender D&I climate and employees’ hope on work depends on employees’ gender such that the positive effect of gender D&I climate is greater on female employees than male employees.

**FIGURE 1:**
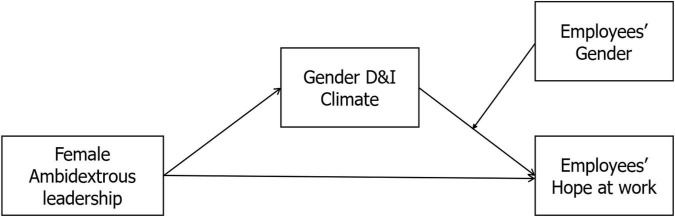
Hypothesized research framework.

This develops the following research model. Figure 1 illustrates the hypothesized research framework.

## Materials and Methods

In this study, quantitative longitudinal survey data was collected from employees working for manufacturing companies in Japan. Initially, the study targeted large manufacturing companies with over 300 employees; however, the criteria should be revised because it was difficult to find participants working in large manufacturing companies in Japan whose direct supervisor is a woman. Therefore, the bar was lowered and participants whose companies employ more than 100 people joined. The data collection was conducted in collaboration with an established survey agency in Japan. Two surveys were conducted, with a two-week gap between them to determine the causal effect of leadership on employees and also to minimize common method bias (MacKenzie and Podsakoff, 2012). Before the first survey, a screening test was conducted to select only participants whose direct supervisor is a woman. Direct supervisor was defined as the leader who the participants contact, report to, and consult with on a regular basis in their organization. This definition was given to the participants in advance. In the first survey, the participants were asked about the ambidextrous leadership behaviors of their female direct supervisor and their perception about the organization’s gender D&I climate. The second survey asked about the participants’ hope on work.

From the two surveys, 311 responses were collected. After removing five survey responses that had the same answers for all questions, 306 responses remained for the analysis. Of the remaining respondents, 156 were men and 150 were women. The average age of the respondents was 43.8 years old. The majority held employee-level positions (183, 59.8%), followed by assistant manager positions (69, 22.55%). Most respondents were highly educated; 206 (67.32%) were graduates of a four-year university program.

## Measures

*Ambidextrous leadership* was measured using the 14-item scale developed by Rosing et al. (2011). The scale describes leaders’ opening and closing behaviors. Sample questions relating to leaders’ opening behaviors are “My direct supervisor gives possibilities for independent thinking and acting” and “My direct supervisor allows different ways of accomplishing a task.” Sample questions relating to leaders’ closing behaviors are “My direct supervisor establishes routines” and “My direct supervisor monitors and controls goal attainment.” Survey respondents were asked to answer each question by thinking about the frequencies of behaviors exhibited by their leaders using a five-point Likert scale ranging from 1 (almost never) to 5 (almost always). The Cronbach’s alpha for the ambidextrous leadership is 0.93.

*Gender D&I climate* was measured using questions developed to represent the concept due to the unavailability from prior studies and the concept’s contextual dependency. Referring to prior research on diversity climate (Pugh et al., 2008; Newman et al., 2018) and interviews with Japanese employees, questions relating to fair and equal treatment of the genders and female career support and advancement in organizations were developed. The five questions are “My company has quite a number of female managers,” “My company has diversity practices to support female employees,” “My company motivates female employees to work better,” “My company treats employees fairly regardless of gender,” and “My company is a good place for women to work.” This concept was rated using a five-point Likert scale ranging from 1 (strongly disagree) to 5 (strongly agree). The Cronbach’s alpha for this scale is 0.85, showing the internal consistency of the questions used in the study.

*Hope on work* was measured using the 6-item scale developed by Luthans et al. (2007). This scale was adapted from the questions developed by Snyder et al. (1996). The questions were intended to measure how hopeful respondents feel about achieving their work goals by developing flexible ideas. Sample questions are “At the present time, I am energetically pursuing my work goals” and “If I should find myself in a jam at work, I could think of many ways to get out of it.” The items were rated on a six-point Likert scale ranging from 1 (strongly disagree) to 6 (strongly agree). The Cronbach’s alpha for this concept is 0.92.

*Control variables* were added to the analysis; respondents’ demographic variables were included in the models. Gender was dummy coded as 1 for the male participants and as 0 for the female participants. Age was a categorical variable coded from 1 to 5 (1 for the 20s to 5 for the 60s), and education was coded as 1 to 5 (1 for high school graduates, 2 for graduates of a two-year college program, 3 for graduates of a four-year university program, 4 for graduates of a master program, and 5 for graduates of a doctoral program). Position was coded as 1 to 5, with a high number indicating a high position. Organizational tenure was measured as the total work period expressed in months. Leader tenure was measured as the period of time working with the current female leader expressed in months.

## Results

Confirmatory factor analysis (CFA) was conducted to ensure distinctiveness among the variables, and the model fit of a three-factor model was compared with that of nested models. The goodness of fit indices of the three-factor model confirmed that it fits the model and that the three variables are distinctive [comparative fit index (CFI) = 0.891, tuker-lewis index (TLI) = 0.880, root mean square error of approximation (RMSEA) = 0.066, standardized root mean square residual (SRMR) = 0.061]. Table 1 presents the mean values, standard deviations, and correlations of the variables. Significant correlations were found among the main independent, dependent, and mediating variables. Female ambidextrous leadership is significantly correlated with gender D&I climate (*r* = 0.37, *p* < 0.01) and employees’ hope on work (*r* = 0.24, *p* < 0.05). Gender D&I climate is also significantly associated with hope on work (*r* = 0.29, *p* < 0.01).

**TABLE 1:** Descriptive statistics among variables (*N* = 306).

	Mean	SD	1	2	3	4	5	6	7	8	9
1. Gender	0.51	0.50	1								
2. Age	2.96	1.07	0.56**	1							
3. Education	3.14	0.63	0.19**	–0.01	1						
4. Position	1.68	1.02	0.34**	0.31**	0.17**	1					
5. Total tenure	5.19	0.84	0.40**	0.75**	–0.06	0.29**	1				
6. Leader tenure	3.32	1.08	–0.06	–0.01	–0.02	0.11	0.10	1			
7. Ambidextrous leadership	2.94	0.85	0.12*	0.04	0.07	0.03	0.03	–0.03	1		
8. Gender D&I climate	3.09	0.84	0.12*	0.11	0.03	0.09	0.07	−0.15**	0.37**	1	
9. Hope on work	3.44	1.03	0.22**	0.32**	0.14*	0.25**	0.29**	–0.00	0.24**	0.29**	1

*Gender: male = 1, female = 0; Tenure: unit = ln(month).*

*Total tenure: the total period of working to date; Leader tenure: the period of time working with the current leader.*

**p < 0.01, *p < 0.05.

Table 2 shows the results of the hierarchical regression analysis of the variables. Model 1 included only the demographic variables and the finding shows that the included six demographic variables explain 14.8% of total variance of hope on work (R-squared = 0.148, F value = 8.663^**^). Specifically, it shows that employees’ age is related to hope on work (β = 0.195, *p* < 0.05). In addition, education and position have significant effects on employees’ hope on work (β = 0.197, *p* < 0.05; β = 0.146, p < 0.05, respectively).

**TABLE 2:** Regression analysis among the variables, female ambidextrous leadership, gender D&I climate, and hope on work (*N* = 306).

	Model 1	Model 2	Model 3	Model 4	Model 5
	Hope on work	Hope on work	Gender D&I Climate	Hope on work	Hope on work
Constant	1.396**	1.458**	0.296	1.388**	1.468**
Gender	–0.020	–0.073	0.033	–0.081	–0.103
Age	0.195*	0.200*	0.036	0.192*	0.198*
Education	0.197*	0.178*	–0.018	0.183*	0.181*
Position	0.146*	0.148*	0.059	0.134*	0.126*
Total tenure	0.133	0.130	0.014	0.127	0.106
Leader tenure	–0.024	–0.018	–0.116**	0.009	0.016
Ambidextrous Leadership		0.266**	0.358**	0.182**	0.181**
Gender D&I Climate				0.234**	0.126
GenderXGender D&I Climate					0.235
R-squared	0.148	0.195	0.171	0.226	0.235
Adjusted R-squared	0.131	0.177	0.152	0.205	0.211
ΔR-squared	–	0.047**	–	0.031**	0.009
F-Value	8.663**	10.340**	8.812**	10.830**	10.080**

*Gender: male = 1, female = 0, Tenure: unit = ln(month).*

*Total tenure: the total period of working by now, Leader tenure: the period of working with the current leader.*

**p < 0.01, *p < 0.05.

Female ambidextrous leadership was incorporated into Model 2, and the finding showed that it has a significant positive effect on employees’ hope (β = 0.266, *p* < 0.01), explaining an additional 4.7% of the total variance in hope toward work [ΔR-squared (Model 2-Model 1) = 0.047]. The significant change in the model fit confirmed the significance of the variable on the dependent variable [change in ΔF (degree of freedom) = 17.530 (1, 298), *p* = 0.000]. This result supports Hypothesis 1. To determine the mediating effect of gender D&I climate, female ambidextrous leadership was regressed on gender D&I climate in Model 3. The results show that female ambidextrous leadership influences gender D&I climate positively (β = 0.358, *p* < 0.01). Model 4 was tested by adding D&I climate to Model 2, and the results show that the significant positive effect of female ambidextrous leadership on employees’ hope on work in Model 2 decreased considerably in Model 4 (from β = 0.266, *p* < 0.01 in Model 2 to β = 0.182, *p* < 0.01 in Model 4), which suggests the potential of the mediating role of gender D&I climate. Sobel (1982)’s test results indicate that the mediating effect of gender D&I climate is significant (*Z* = 3.388, *p* < 0.01). In addition, the bootstrapping technique suggested by Preacher and Hayes (2004) was employed to reconfirm the significance of the mediation effect. The test results indicate that the mediation effect of gender D&I climate on the link between female ambidextrous leadership and employees’ hope on work is significantly valid (β = 0.102, *p* < 0.01; bootstrap normal-based 95% confidence interval (CI) [0.041, 0.176]). Therefore, hypothesis 2 that assumed the mediating role of gender D&I climate between female ambidextrous leadership and employees’ hope on work is supported. Model 5 added the interaction term of gender and gender D&I climate to test the moderating effect of gender on the relationship between gender D&I climate and hope on work. Hypothesis 3 assumed that gender D&I climate is more critical for female employees than male employees; therefore, the positive effect of gender D&I climate on employees’ hope would be more considerable among female employees than among their male equivalents. However, Model 5 uncovered a non-significant moderating effect of gender (β = 0.235, n.s.), and the interaction term (Gender X Gender D&I climate) explains only 0.9% of the total variance in the dependent variable [i.e., hope on work; (ΔR-squared (Model 5-Model 4) = 0.009]. The non-significance of the value change in model fits also indicated that the moderating term does not determine the dependent variable [change in ΔF (degree of freedom) = 3.426 (1, 296), *p* = 0.065]. This finding means that no support was derived for Hypothesis 3.

## Discussion

The study investigates Japanese employees who work with female supervisors and examines the relationships among the ambidextrous leadership of female supervisors, gender D&I climate, and employees’ hope on work. Female leadership studies have focused only on examining the perceptions and appraisals of female leadership and the potential organizational benefits of the increases in female leaders (Eagly and Carli, 2003; Stoker et al., 2012; Girdauskiene and Eyvazzade, 2015; Szymanska and Rubin, 2018). However, the prior studies ignored the specific and qualitative aspects of female leadership, such as its characteristics and the underlying mechanism through which employees are influenced. Addressing this gap, this study focuses on the ambidextrous leadership style of female leaders and its effect on shaping organization’s gender D&I climate and ultimately its influence on employees’ hope on work. This first empirical findings contribute to enhance understanding of the effectiveness of female leadership style vis-à-vis employees’ perception of organizational environment and their psychological state regarding their work in Japan.

An interesting and unexpected finding should be addressed. The study assumed that the effect of pro-gender D&I climate on hope would be greater among female employees than male employees (Hypothesis 3). Contrastingly, the results showed a non-significant effect of gender on the relationship between gender D&I climate and employee hope—a phenomenon that contradicts the view advocated in social identity theory (Tajfel and Turner, 1986; McKay et al., 2008). A possible explanation for this result lies in the diversity of individual social identity, which thus means that the extent to which a certain social identity is shaped depends on individuals (Newman et al., 2018). In the same vein, the recognition of female identity by women varies per person. Therefore, the extent to which gender D&I climate influences on female employees is contingent on their level of female identity. That is, females who have strong female identity are more remarkably influenced by gender D&I climate than those with low female identity. Such individual differences in female identity among female employees might draw such result.

Another reason for the above-mentioned result is that gender D&I climate is an immediate and relevant issue not only to female employees but also to their male counterparts. Pro-gender D&I climate signifies an organization’s choice to treat employees fairly by fostering an open and flexible organizational climate, in which differences in ideas and needs among employees are allowed and accepted. Such a climate is therefore meaningful to all employees; that is, it exerts the same effects, with no considerable differences, whether an employee is female or male.

### Theoretical and Practical Contributions

The significance of this research should be addressed. First, this study contributes to advancing the prior female leadership studies (Girdauskiene and Eyvazzade, 2015; Miyake, 2015; Szymanska and Rubin, 2018; Takada, 2009). In particular, this is the first attempt that disclosed the effectiveness of female ambidextrous leadership in Japan. The study highlights that female ambidextrous leadership sends a positive signal about companies’ gender D&I management, helps shape the perception of pro-gender C&I climate among employees, and consequently, increases employees’ hopeful and positive energy regarding their works. This finding lends a support of ambidextrous leadership theory (Rosing et al., 2011) and social information processing theory (Salancik and Pfeffer, 1978). Considering the paucity in empirical evidence of female leadership effectiveness and its underlying process, particularly in Japan, the findings of showing the positive and procedural effect of female leadership on employees’ hope on work have a particular academic value, contributing to the development of female leadership studies.

In addition, the study contributes to the understanding D&I management in the HRM studies (McKay et al., 2008; Newman et al., 2018). As pointed by Farndale et al. (2015), the diversity concept and relevant diversity issue vary across counties. The broad-defined concept of diversity prevalent in the West is not applicable in Japan because of its specific socio-contextual characteristic. Therefore, this study focused on one aspect of diversity, gender D&I that is the most relevant to the Japanese context. Referring to prior studies (Pugh et al., 2008; Newman et al., 2018) and interviewees’ responses regarding their perceptions on companies’ gender D&I climate, the study developed the five-item scale measuring gender D&I climate. This context-contingent approach in D&I management can capture and explain better the effect of diversity management. Also, the context-contingent approach with a focus on gender D&I can be applied to other Eastern contexts such as South Korea in which gender D&I is the most relevant and immediate diversity aspect as similar as Japan. Taking the context-contingency view, this study broadens the perspective understanding D&I management, contributing to developing the current D&I management studies.

Related to this, given that human resources are a valuable source of organizational growth and sustainability, the finding regarding the positive effect of gender D&I climate on employees’ hope has a significant implication. Organizations’ initiatives in terms of gender D&I and shaping gender D&I climate indicate they are making an effort to treat their employees fairly and equally. Such efforts are favored by employees; thus, employees show optimistic and hopeful attitudes at their work. This psychological process implies that organizations’ stance about gender D&I management signaled by female ambidextrous leadership shapes pro-gender D&I climate, which thus is linked to the increased work motivation of employees. More importantly, the lack of moderation from gender on the effect of gender D&I climate on employees’ hope reflects that such a positive effect of pro-gender D&I climate on shaping employees’ positive work attitudes is not confined to a particular gender. In other words, favorable perceptions about D&I practices and management in Japanese companies can benefit and increase all employees’ hope, regardless of gender. The finding further signifies that Japanese employees are conscious of and favor D&I management. If they believe the climate in their organizations to be gender inclusive, they are more motivated and engaged in their work. Therefore, companies should actively implement gender D&I management to shape a climate that favors gender D&I. Such efforts not only increase the psychological capital of employees but also enhance their productivity and financially benefit companies (Ali, 2016). In clarifying the direct influence of gender D&I climate on employees’ hope, the study contributes to the development of prior diversity management studies in Japan (Miyake, 2015; Takada, 2009).

Expected practical implications are also worth noting. First, the findings may motivate Japanese companies to embrace and cultivate female leaders and welcome the advantages of implementing gender D&I management. Japan has a strongly stereotyped view of gender roles and a firm belief in role congruity, with leadership seen as equivalent to direction by males (Eagly and Karau, 2002). This stereotype and the reluctance to initiate changes have delayed the implementation of gender D&I management in Japanese companies. This delay, in turn, has given rise to explicit and implicit disadvantages for women, discouraging female talents from aspiring for career advancement. The upshot of all these is the loss of company competitiveness. The results of this study can serve as reference for Japanese companies that want to eliminate the long-standing social misunderstanding of gender roles and concerns about female advancement. The finding on the positive effects of female leadership on employees’ work motivation and energy can convince Japanese companies that it is advisable to embrace gender diversity and that they can expect potential benefits from this initiative. The acceptance of gender diversity shapes institutional norms regarding gender D&I management in Japan. As the institutional normalization of gender diversity facilitates its direct and indirect financial benefits by assuring stakeholders of the advantages of such management (Zhang, 2020), industry- and society-wide social acceptance is expected to bear fruit for Japanese companies who engage in gender D&I actively.

The findings can also be useful in the leadership development of Japanese companies. The results indicated that the ambidextrous leadership skills of female leaders enable them to effectively increase perceptions regarding on pro-gender D&I climate and enhance employees’ hope on work. This finding can be utilized to companies to identify, educate, and train female employees for leadership positions. Focusing on the ambidextrous nature of leadership, companies can find female employees who are capable of exhibiting such leadership behaviors or they can foster such leadership skills of potential leaders by designing and providing leadership training programs. Such efforts will benefit companies to achieve diversity management effectively and thus draw successful and sustainable outcomes from it.

### Limitations and Future Research

Despite the aforementioned implications, the study has some limitations. First, the study only focused on the ambidextrous behaviors associated with female leadership. Even if the findings indicate that female leaders exhibit such ambidextrous leadership behaviors and that such leadership influences employees positively, the study did not consider other female leadership styles. Prior studies revealed that female leaders exert transformational behaviors more than male leaders and also, such female transformational leadership is as effective as male leadership (Eagly et al., 2003; Stempel et al., 2015; Kim and Shin, 2017). Therefore, future studies should investigate other female leadership styles together with ambidextrous female leadership. By comparing and contrasting different female leadership styles, future studies can clarify the exact attributes of female leadership and the effects on employees.

Second, this study investigated only employees’ hope on work as a consequence of female leadership. However, other work-related concepts, such as work commitment, psychological empowerment, organizational citizenship behavior, and innovative behavior, may be associated with female ambidextrous leadership. Considering the potential relationship between female ambidextrous leadership and other positive attitudinal and behavioral concepts, future studies should examine other potential target variables that female ambidextrous leadership may influence. Adding these variables and determining their relationship with female ambidextrous leadership will enhance the understanding of this leadership style and its potential and practical impact on employees.

Related to this, the research model may be inapplicable to other research contexts. The study showed that direct ambidextrous leadership by females denotes organizational diversity management and shapes employees’ perception of pro-gender D&I climate in organizations. This indicates that female leaders serve as change agents of organizations, not merely cogs in a machine (Szymanska and Rubin, 2018). However, depending on the social cognition of gender in organizations and general viewpoints regarding female leadership, this research model may or may not be supported. Therefore, to generalize the research model, it necessitates investigating female leaders’ roles, along with general perceptions and evaluations of female leadership in the future studies.

Finally, this study did not control for organizational factors. Given that the attitudes of individuals about their organizations and jobs are influenced by socio-environmental factors, researchers should explore other social and environmental determinants, such as the quality of the relationship between employees and their female supervisors, organizational structure, organizational support, and work design, as these may influence how employees view their organizations and shape their attitudes toward work. Future studies should incorporate and regulate the potential effects of such factors to validate and generalize the research model put forward in the current work.

## Conclusion

Japan, the homogenous and aging society is facing the shortage in their workforce, which diminishes their business competitiveness. One effective solution that Japanese companies can choose may be to engage in and implement gender D&I management effectively. Focusing on the significant meaning of gender D&I in Japan, this study addresses this issue. Building on social informational processing theory and social identity theory, the study examined female ambidextrous leadership’s effect on employees’ hope on work and its underlying mechanism. Findings showed that female ambidextrous leadership strengthens employees’ perception on pro-gender D&I climate of their organizations. This improved perception of pro-gender D&I climate contributes to increase employees’ positive and hopeful energy at work. In addition, the positive effect of pro-gender D&I climate on employees’ hope is the same for all employees regardless of gender. The findings highlight the effective female leadership style and its positive effect on shaping employees’ positive work attitude. Therefore, the study contributes to the development of female leadership and diversity management studies in Japan. In addition, the study provides empirical evidence of the positive effects of female leadership on employees and highlights the potential benefits of gender D&I by advancing and cultivating female leaders in Japanese companies.

## Data Availability Statement

The raw data supporting the conclusions of this article will be made available by the authors, without undue reservation.

## Ethics Statement

Ethical review and approval was not required for the study on human participants in accordance with the local legislation and institutional requirements. Written informed consent for participation was not required for this study in accordance with the national legislation and the institutional requirements.

## Author Contributions

The author confirms being the sole contributor of this work and has approved it for publication.

## Conflict of Interest

The author declares that the research was conducted in the absence of any commercial or financial relationships that could be construed as a potential conflict of interest.

## Publisher’s Note

All claims expressed in this article are solely those of the authors and do not necessarily represent those of their affiliated organizations, or those of the publisher, the editors and the reviewers. Any product that may be evaluated in this article, or claim that may be made by its manufacturer, is not guaranteed or endorsed by the publisher.

## Footnotes

1 The term was first introduced in a Goldman Sachs’ research report titled “Womenomics: Buy the Female Economy” (Matsui et al., 1999). The research shows that female participation could contribute significantly to economic growth in Japan. The research was continued, and the recent “Womenomics 5.0” report argues that resolving the gender gap in employment could increase Japan’s GDP by 10% and that if the ratio of female to male working hours rose to the average OECD level, Japan could achieve a 15% increase in GDP.
